# Community Nurses’ Perceptions of Their Role as Health Promoters in Collective Prevention: A Qualitative Study in Dutch Community Nursing Care

**DOI:** 10.1177/21501319261445207

**Published:** 2026-05-05

**Authors:** Sara Francina Mirjam de Leede-Brunsveld, Geertuida Johanna (Truus) Teunissen, Jolanda Lindenberg, David van Bodegom, Johanna Adriana Henrica (Anneke) van Vught

**Affiliations:** 1 Leiden University Medical Center, The Netherlands; 2 Leyden Academy on Vitality and Ageing, The Netherlands; 3 University of Humanistic Studies, Utrecht, The Netherlands; 4 Dutch Healthcare Authority, Utrecht, The Netherlands; 5 Radboud University Medical Centre, Nijmegen, The Netherlands

**Keywords:** community nursing, collective prevention, qualitative research, population health, prevention, community health, health promotion, qualitative methods, lifestyle change

## Abstract

**Introduction:**

Addressing the demands of an ageing population requires a shift from individualised to collective prevention. Collective prevention adopts a socio-ecological approach, mobilising communities to improve population health. This study explores community nurses’ (CNs) perceptions of their role as health promoters.

**Methods:**

A qualitative study using participatory action research was conducted among 58 CNs from 18 community nursing care teams over 3 years. Data collection included 11 focus groups, a World Café session, and participant observations. Data were analysed through iterative thematic content analysis.

**Findings:**

From the analysis, 3 themes emerged: (1) CNs understanding of prevention, (2) their views on their roles and competencies, and (3)what are the facilitating and hindering factors to implement collective prevention. CNs’ understanding of prevention evolved from individual “aversion” of disease to a community-based vision centred on social connection. Four key roles for CNs were identified: detector, motivator, facilitator, and organiser. While a shared vision within teams facilitated progress, significant barriers included time pressure, productivity-based funding, and a lack of training in nursing curricula.

**Conclusion:**

CNs role in collective prevention is important, transitioning from task-oriented nursing care to community empowerment. However, sustainable implementation requires integrating collective prevention into national policy funding, and nursing education.

## Introduction

The Netherlands, like many other countries, is facing major challenges in organising sustainable healthcare for its ageing population. To maintain healthcare accessibility given increasing demand and workforce shortages, a greater emphasis on prevention is essential to reduce ill health, associated burdens, and costs.^1,2^ A proactive strategy to achieve this includes strengthening health protection, health promotion, and disease prevention, which can significantly improve both individual and population health and well-being.^3^ Each of these 3 forms of prevention has a particular focus. Health protection aims to shield individuals from health risks, health promotion empowers people to gain greater control over their health by adopting healthier lifestyles, and disease prevention targets the reduction of risk factors and the halting of disease progression.^3,4^ Nurses are trained as health promoters and can play a key role in implementing these strategies, particularly in public health, primary care, and community- and home-based settings.^5,6^ However, nursing-led health promotion efforts have largely focussed on individual prevention,^7^ including interventions such as lifestyle counselling, motivational interviewing, and fall risk assessments. While valuable, these approaches primarily aim to influence individual behavioural change even though lifestyle-related ill health and disease are widespread in the population.^8,9^ An alternative approach to promoting healthy behaviour is through collective prevention; see Box 1 for an explanation of collective prevention.

**Box 1.** Description of Collective Prevention.^10–13^

Collective prevention is a group- based approach. The focus shifts from an individualised, to support healthy behaviour towards a group-based, approach. Collective prevention brings individuals together, not necessarily based on their personal risk profiles, but by identifying their common problems or shared risks. Collective prevention is rooted in a socio-ecological model of health, which places emphasis on the social, societal, and physical environments and the roles these play in health outcomes. These environments and adopting a contextual approach may form an integral part of preventive activities. It is important that community members are actively engaged in preventive efforts, co-develop preventive initiatives, fostering a sense of ownership in addressing health challenges. These include activities designed to promote health, prevent health problems, or limit the impact of a disease.

Collective prevention should not be confused with community-based prevention. While both approaches involve local populations, the distinction lies in their orientation. Community-based prevention often targets defined geographic areas, such as neighbourhoods, with externally designed interventions. In contrast, collective prevention focusses on mobilising communities (regardless of their geographic or cultural form) to assume agency and responsibility in health promotion and prevention efforts.^14^

Historically, Dutch community nurses (CNs) developed their role as health promoters in close collaboration with social work. However, from the late 1990s onwards, the rise of product-orientated thinking in Dutch healthcare led to a more medicalised and task-focussed approach.^15^ This shift narrowed the scope of community nursing care, prioritising individualised care driven by diagnoses and standardised procedures. Consequently, the holistic, community-oriented aspects of the nursing role (such as collective, supportive, and rehabilitative prevention) have gradually diminished.^16,17^ Community nursing care takes place primarily at the homes of clients and is provided alongside other healthcare professionals such as the general practitioner and other (paramedic) professionals in primary care.^17^ Despite growing interest, little is known about the perspectives of community nurses (CNs) on collective prevention in practice.^18^ Therefore, we studied the role and perspectives of CNs in a large collective prevention project in the Netherlands. Buurtzorg, a Dutch community care organisation, launched a collective prevention programme in 2018. This project was inspired by principles integrated into the Cuban health system.^19-21^ The Cuban model emphasises collective rather than individual prevention, particularly for older adults, through neighbourhood initiatives such as *círculos de abuelos*, which encourage physical activity, social cohesion, and mental well-being.^22^ In this context, CNs are key agents in mobilising and facilitating participation.^19^ Currently, 28 Buurtzorg teams throughout the Netherlands implement collective prevention initiatives. The approach begins with group-building activities to build trust and connection, which evolve into health-protective, health-promotion, and disease-prevention-focussed programmes such as diabetes lifestyle groups, walking clubs, and *Vitality Clubs.*^19,23^ These programmes have the potential to not only support physical health but also contribute to social connectedness and mental well-being.^24^

This study explores the perceptions of CNs, defined here as their experiences, beliefs, and attitudes, regarding their role as health promoters within collective prevention. Gaining further insight into CNs’ perspectives, particularly given the limited empirical research on collective prevention conducted by CNs,^25^ can help further implement collective prevention in community nursing care.

## Methods

### Study Design

The qualitative research we report on in this article is part of a larger multi-method study. A participatory action research design fitted the need for both responsiveness and a contextual approach.^26^ This allowed us to use research methods and formats throughout the study that suited the process. We continually discussed how we could best gain insight into the implementation of collective prevention and the role of the CNs within this. In this article we focus on the qualitative data of the study to explore how the participants see the meaning of their role as health promoters in collective prevention in community care.^18^

### Study Participants and Setting

This study was conducted among CNs working in community nursing care teams. The CNs are from 18 different teams from across the Netherlands who self-selected to participate in a programme to develop and implement collective prevention in the community care organisation Buurtzorg^18^ (see Box 2). CNs in this study are interns (EQL 4 and EQL 6), nurse assistants, registered nurses, district nurses (DNs), and nurse practitioners (NPs). All participants were selected based on purposeful sampling.^27^ All teams had agreed to participate in the research project.^18^ Each individual team selected a project leader to coordinate the collective prevention project within their team. They were, most of the time, the so-called driving force of the collective prevention project in their team. Each participating team received an invitation and decided themselves who they would send to the focus group.

**Box 2.** Buurtzorg Approach.^28^

The Buurtzorg approach start from self-organising, small teams (with a maximum of 12 nurses per team) with a focus on client independence, prioritising people, and minimising bureaucracy. The Buurtzorg model forms the foundation of the community care practice, with the client in the centre, with around the client the informal network. The Buurtzorg team is around the client and the informal network. At the outside is the formal network such as other healthcare professionals. They all are collaborating with the client and their social environment. Communication, flexibility within the teams, and the use of technology for knowledge sharing are crucial to this approach.

### Study Procedures

The study followed an iterative 3-phase design, a structure informed by Participatory Action Research (PAR) principles.^26^ The transition between these phases was a pragmatic choice, aimed at increasing the depth of the data through continuous refinement and verification.

- Phase 1: Exploration. This phase focussed on identifying the primary themes and experiences within the teams.
- Phase 2: Deepening. To achieve greater depth, findings from phase 1 were presented to a combined group of CNs who participated in the research and some who did not participate. This allowed for the refining and testing of initial insights by bringing different perspectives together, ensuring the data moved beyond observations.
- Phase 3: Validation. The final phase focussed on verifying the findings. Using a World Café format, the refined insights were presented to CNs who participated in the collective prevention projects to ensure the final conclusions accurately reflected the reality of the practice.

The approach to participant recruitment varied across the phases (see also Table 1).

**Table 1.** Overview of the Number and Duration of the Focus Groups.

Phase	Number of focus groups	Duration	Number of participants	Topic list
1: Sept 2022-March 2023	4 in-person focus groups	Range: 83-115 min	37	Appendix 1
2: February 2024	5 in-person focus groups	Range: 75-96 min	26	Appendix 2
2: April 2024	2 online focus groups	Range: 75-76 min	9	Appendix 3
3: November 2024	World café	105 min	12	Appendix 4

- All phases: participant observation through field visits. We started our study by contacting each team every 3 months in person, via email, and/or by phone by the researchers (between 3 and 10 times per year). In addition, each team was visited at least once, up to 3 times, during the study. During these visits, researchers had informal, unstructured conversations with the team members and participating community members. They joined in the activities, such as walking groups, exercise groups, and lifestyle support groups.
- Phase 1: All teams who participated in the research received an open invitation to participate in a focus group via email.^27^ In total, 39 participants were invited, and 37 attended 4 focus groups (range n = 8-12); 2 participants withdrew due to personal reasons.
- Phase 2: All teams were invited to focus groups for the refining and testing. In total, 42 people were approached, and 35 attended; 7 participants dropped out: 4 because their team had been disbanded and 3 individuals were unable to attend due to work demands.
- Phase 3: Project leaders from each participating team were emailed and asked if one representative from each team could attend the validation session for member checking. In total 12 CNs attended the World Café from 9 different teams.

To ensure methodological consistency and rigour, the focus groups and validation sessions were facilitated by different members of the research team.

### Data Collection

Participant observation: following the PAR methodology,^26^ researchers conducted participant observations during field visits to the participating teams. These observations and informal, unstructured conversations focussed primarily on the CNs to gain insight into their professional activities and team dynamics. While community members were present in these settings and informal interactions took place, the data discussed in this article was limited to the experiences and actions of the CNs. Field notes and photographs were used to document the context of the collective prevention interventions. This approach was fully covered by the ethics approval obtained for the overarching project.

Focus groups were held to encourage interactive discussion and to gain a broad range of perspectives among the CNs, a strategy that aligns with the collaborative nature of PAR. In contrast to individual interviews, this format allowed for the exploration of experiences and challenges. The design and moderation of these sessions were informed by the principles of focus group interviewing.^29^

To ensure consistency across the different sessions, a focus group guide (see Appendices 1–4) was developed and shared with participants beforehand, facilitating individual reflection prior to the group discussion. All focus groups were audio- and video-recorded with the permission of the participants. 9 out of 11 focus groups were held in person. Two focus groups were conducted online; most of these participants came from various parts of the Netherlands and had limited time to travel.

World café: In the third phase, we did one session using the World Café Method. A World Café^30,31^ typically involves a series of small group discussions, followed by a report-back session where the main themes and insights are synthesised and reported. The group was divided into 3 smaller groups (N = 4), with each group assigned a topic for discussion and creative expression. The topics were chosen after analysing the data from the 11 previous focus groups. After 12 min, participants rotated to another table to engage with a different topic. After 3 rounds, a summary (15 min in total) of each group’s input was presented by the researcher. The World Café method was audio recorded, and pictures were taken by the first author from the input that was made by the participants. These pictures are used in this article for illustration and better understanding of what has been said.

### Data Analysis

Nine focus groups were transcribed verbatim by a professional transcription service after signing a data processing agreement. After AI transcription was approved by the host institute and blinded for review, the last 2 focus groups were transcribed verbatim by the first author with the support of Amber Script.

The study employed thematic analysis,^32^ using an iterative and constant comparative approach. The analysis proceeded through several stages:

1. Initial coding: The first author developed initial semantic codes based on a subset of the data.
2. Refinement: These codes were discussed and refined through peer debriefing with the research team.
3. Thematic mapping: A coding tree was developed to analyse the relationships between codes, moving the analysis from a content-driven level to deeper thematic patterns.
4. Final: This iterative process helped identify 3 themes which were reviewed and finalised by the entire research team.

### Ethical Considerations

This study was reviewed and determined not to fall under the scope of the Dutch Medical Research Involving Human Subjects Act by the Institutional Review Board of the Medical Ethical Committee for observational studies.

Participants received both oral and written information about the study, including details of confidentiality. They were asked to sign the informed consent and return it to the first author. They were informed that participation was voluntary and that they could withdraw their consent at any time. Additionally, they were notified that recordings would be transcribed and that video footage might be reviewed to clarify the speaking. All results are presented anonymously using a number for each participant.

All focus groups were recorded and securely stored by the first author on a secured drive, protected with a password and authenticator, in accordance with the ethics protocol. This also applied to the material collected during the field visits and the validation session. All data collected will be reported anonymously in publications.^33^

### Reflexivity and Rigour

The researchers collaborating in the study had prior experience conducting research with professionals working with older populations and had backgrounds in healthcare research and work. The first author, a PhD candidate, is a nurse practitioner with a master’s degree and is also employed by Buurtzorg. The dual role of the first author as both a researcher and an employee could potentially compromise objectivity and the openness of the participants. Yet her familiarity with the organisation’s working methods may also facilitate a deeper understanding of certain statements made by participants. To mitigate potential bias, continuous feedback and discussions were held within the research team to ensure accurate interpretation. This approach required a reflective attitude, maintained by encouraging self-awareness of potential biases and preconceptions during team discussions.

Rigour and trustworthiness were ensured using the criteria of credibility, dependability, confirmability, and transferability.^34^

- Credibility: This refers to the “truth value” of the findings. Credibility was ensured through prolonged engagement; the first author visited all teams participating in the research several times, building trust and gaining a deep understanding of the CNs’ context. We used investigator, data, and method triangulation^35^ (merging insights from focus groups, the World Café, observations, field notes, and photographs) to increase the validity of the findings. Furthermore, member checks were conducted in December 2023 and November 2024, during which emerging interpretations were discussed with CNs to obtain their reactions and further insights.
- Dependability: This involves the stability of data over time and under different conditions. Rigour was established through triangulation to achieve a high degree of data density. In line with the aim of the overarching study, we sought to document the evolution of perspectives over time, acknowledging that during the study period, new insights emerge as participants learn, making traditional saturation less applicable than data density.^18^ Peer debriefing within the research group further ensured that the research outcomes were in line with the data collected.
- Confirmability: This ensures that the findings are shaped by the participants and tries to limit researcher bias. Confirmability was assured by maintaining a journal throughout the study to document the first author’s perspectives. Additionally, we used investigator triangulation through the participation of 4 researchers in data collection (in changing pairs) and analysis. All codes and themes were cross-checked within the research team to verify the findings. This article followed the 32-item COREQ checklist to ensure comprehensiveness and transparency.^36^
- Transferability: This refers to the degree to which findings can be applied to other contexts. We provide thick descriptions of the study procedures, the role of the CNs, and the specific organisational setting to allow readers to assess the applicability and comparability of these findings to similar community nursing environments.

## Findings

### Characteristics of Participants

In Table 1 we give an overview of the community nurses (CN) who participated in this study. They include interns, nurse assistants, registered nurses (RNs), district nurses (DNs), and nurse practitioners (NPs), all working for Buurtzorg. They are from 18 different teams from across the Netherlands. Most of the participants are female. Most of the CNs who participated in the research project have over 20 years of working experience, and they are older than 50 years. Most CNs are working as registered nurses (EQL 4) or as district nurses (EQL 6). Percentages are provided for contextual purposes to illustrate the sample size relative to all employees of Buurtzorg, rather than to suggest statistical generalisability (Table 2).

**Table 2.** Demographic Characteristics of Community Nurses Participating in the Research Project and All Employees of Buurtzorg Netherlands.

Characteristic of community nurses	Sampled employees (N = 58)	All employees (N = 7536)^a^
Gender	N = 58	N = 7731^b^
Male	3 (5%)	313 (4%)
Female	55 (95%)	7418 (96%)
Age	N = 58	N = 7536
<20	0	4 (<1%)
20-29	2 (3%)	1233 (16%)
30-39	5 (9%)	1391 (18%)
40-49	6 (10%)	1137 (15%)
50-59	20 (34.5%)	2102 (28%)
60+	20 (34.5%)	1669 (22%)
Not reported	5 (9%)	
Educational level community nurses	N = 58	N = 7731
Interns	3 (5%)	195 (2.6%)
Nurse assistant EQL (3)	7 (12%)	2114 (28%)
Registered nurse EQL (4)	22 (38%)	3144 (42%)
District nurse EQL (6)	22 (38%)	2252 (30%)
Nurse-practitioner-EQL (7)	4 (7%)	26 (0.4%)
Years of nursing experience	N = 58	Unknown
0-9	5 (9%)	
10-19	7 (12%)	
20-29	10 (17%)	
30-39	13 (22%)	
40+	9 (16%)	
Not reported	14 (24%)	

a Total number of nurses qualified: EQL 3, EQL 4, EQL 6, and EQL 7.

b Including interns.

## Themes

From the analysis, 3 themes emerged: (1) the concept of prevention described by CNs; (2) their views on the roles and competencies needed to act as health promoters in collective prevention; and (3) the factors they experience as either facilitating or hindering collective prevention efforts.

### **Theme 1:** Meanings of Individual and Collective Prevention

#### Prevention

CNs gave a wide range of answers and definitions to the research team when they were asked to describe the term “prevention.” What we observed was that in the first round of focus groups, the term “avert” was used by most participants to describe prevention. With aversion, they meant that they would prevent any potential negative health outcomes. In their descriptions, they mentioned separate phases to prevent adverse health outcomes, to avoid becoming more ill, and to avoid side effects of disease. For instance, pressure ulcers or the risk of getting a disease, such as diabetes. One CN said that prevention is a way of averting and explained the following: “*Yes, physical activity, smoking, alcohol*, *nutrition, and relaxation. For me, those are the traditional prevention topics*.” (Participant 46, DN, phase 1 – exploring).

#### Individual and Collective Prevention

In our analysis, we noticed that CNs initially used common descriptions for prevention that aligned with individual prevention interventions. However, over the course of the research, we observed a shift towards a more group-based approach, looking at common problems or shared risks on a group level and using collective-based interventions. In the first round of focus groups, most CNs explained the term “prevention” in terms of individual prevention, referring to the prevention they do in their daily work when they visit clients at home. They describe prevention as making sure that someone is not becoming more ill or deteriorating due to the consequences of the disease.

It is not always easy. But I do try to bring up prevention early in conversations. For instance, if someone is very short of breath, you might adjust their medication, but at the same time, I explain why it is important to stay active, to keep the circulation going. I always try to weave that into the discussion. (Participant 67, NP, phase 2 – deepening).

What we found is that CNs describe prevention as something they just do. It is a routine intervention applied with clients, carried out automatically, without much conscious reflection. For instance, they may place an anti-decubitus cushion for someone developing early pressure ulcers.

And I find it truly important (and genuinely fulfilling) to feel that I’m not just dealing with symptoms, so to speak, but that I’m also working on the other side, actively preventing things from happening. That gives me a very rewarding feeling. (Participant 44, DN, phase 1 – exploring).

In the second round of focus groups, the participants described prevention more in terms of focussing on group risks in the community, in line with common conceptualisations of collective prevention. Especially the CNs who participated in the research project developed a different understanding of the term “prevention” over time. They explain that prevention can be something carried out collectively within a neighbourhood. “Doing it together” is frequently mentioned in these second focus groups, in contrast to the first round when it was rarely mentioned. CNs describe how joint activities such as walking groups or lifestyle groups bring people together to engage in health-promoting practices instead of them personally visiting and advising individuals. The participating CNs describe that these activities are ideally continued by community members themselves, sometimes with the support of local volunteers. Many such activities have a dual purpose that CNs consider strengthens their effectiveness: for instance, combining physical activity with a social element, such as sharing coffee afterwards, thus addressing both physical and social well-being.

Collective prevention is about connection, about social encounter. (Participant 39, DN, phase 2 – deepening).

#### Health Protection, Health Promotion, and Disease Prevention

CNs started to make a difference in the description of prevention and redefine it over the course of the project. Their understanding came to align more closely with the 3 forms of prevention as described in the literature, namely health protection, health promotion, and disease prevention. CNs describe health protection from a more strategic, population-level perspective to address recurring health issues within a neighbourhood. By identifying patterns among clients living in the same area; such as frequent addiction problems or social vulnerability, CNs working in collective prevention develop interventions that reach the wider community.

If you have many clients in the same neighbourhood, you can identify patterns and move from an individual to a group-level approach. But you need the right conditions: a strong community focus, good local network knowledge, and a clear map of the area and its people. (Participant 66, NP, Phase 2—deepening, April 2024).

Health promotion is seen as preventing disease, decreasing negative health outcomes when being ill, and providing lifestyle advice too. CNs also provide health promotion to caregivers, family members, and/or the client’s wider network. They also gave different examples of whom they give health promotion to. For example, for people with diabetes, they encourage community members to join walking groups to promote physical activity: “*It is also a bit about how you think of yourself or how you approach life. For me, lifestyle is important. But you also want to convey that. In some way, when you are with clients and something comes up, you feel you can share [. . .] maybe eat a bit more of* this or *less of* that or *be more physically active* (things *like that*) it *makes a difference*.” (Participant 42, intern/RN, phase 2 – deepening).

As CNs reflected on their evolving role as health promoters during the World Café workshop in phase 3, they expressed a growing awareness of prevention as a broader concept. It has become a new, collective vision. Many mentioned that through participating in collective prevention initiatives, they had begun to see their work in a new way.

**Figure 1.**
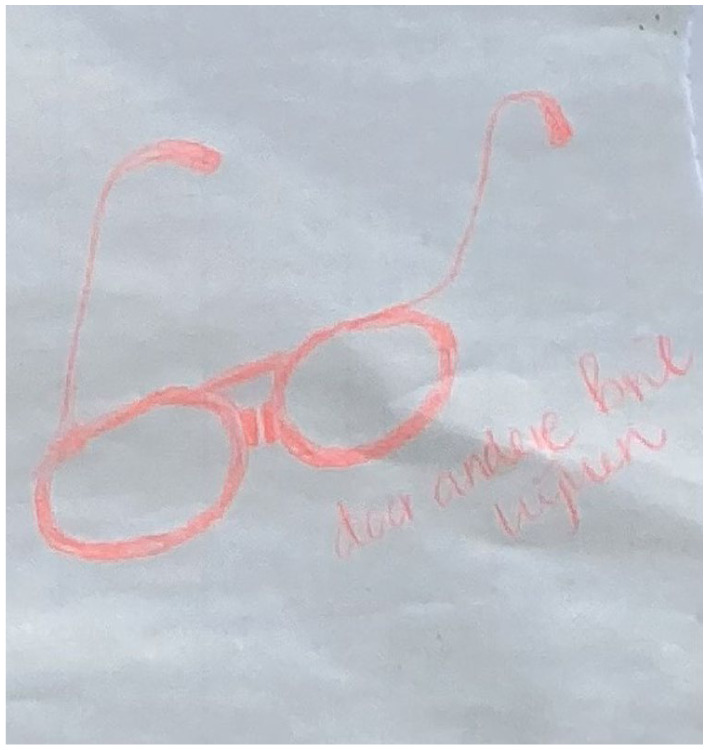
Phase 3 – validation, “Looking through other glasses.”

As illustrated in Figure 1, they described this as “looking through different glasses.” This new perspective focuses on connection, empowerment, and a shared responsibility for health at a group-based level.

#### The Importance of Prevention

When reflecting on the importance of prevention, CNs emphasised that prevention is much more than just preventing illness. It contributes to the quality and meaning of life.

And of course, we applied for the duo bike subsidy together with a community member whose wife was disabled [. . .]. We made a health-promoting intervention, and one colleague went cycling with the community member. The person was absolutely delighted and feeling included. (Participant 58, DN, phase 1 – exploring).

CNs often emphasise prevention’s necessity for the future. They explain that the rising number of people living with chronic conditions who remain at home makes prevention essential. With this increase, CNs highlight prevention not only as necessary but also reiterate policy discourses in seeing prevention also to ease the growing pressure on healthcare professionals and resources:

Prevention is also about stopping people from getting sick in the first place. So many illnesses in the neighbourhood are lifestyle-related; they can be prevented, which means less care is needed. Right now, so many people already work in healthcare, and by 2040 it will have to be even more. That is simply not sustainable. So, prevention is also about making sure we do not collapse under the weight of it all; we need people to stay healthier and to live together differently. (Participant 44, DN, phase 1 – exploring).

In the end, you would have lower healthcare costs. If people stay healthy for longer, they visit the GP less and need fewer medicines, that is the idea behind it. (Participant 61, DN, phase 2 – deepening).

### **Theme 2:** Roles and Competencies as Health Promoters in Collective Prevention

During the 3 years of the programme, our analysis of the data showed that CNs increasingly see themselves not only as care providers but also as health promoters who play a proactive role in addressing health issues at both individual and collective levels. This responsibility comes with a broad range of roles they fulfil, from identifying pressing issues in communities to mobilising residents and coordinating with professional networks. Below we describe these roles in more detail.

#### Detector

The prevention projects were initiated by CNs following a Buurtzorg-provided training session, which included both online and face-to-face workshops, 6 in the first 2 years, then 2 per year. CNs start prevention projects in the neighbourhood with identifying community issues. They begin by mapping out the health landscape of a neighbourhood. This involves both desk research using epidemiological data and direct interaction with community members. Essential parts of this approach are interviewing people at random in the neighbourhood and simply walking around to observe everyday life. The emphasis lies on listening, watching, and identifying what might otherwise remain unnoticed and may not be identified through structured questionnaires. The detector role in collective prevention has a group-based approach. A CN scouts for underlying social, health, and other common problems or shared risks that affect community members who are not yet receiving community care.

Indeed, we conducted research in the community to understand the wishes and needs of local residents. Students helped to complete a large number of questionnaires. (Participant 58, DN, phase 1 – exploring).

Participants identified observational skills as indispensable competencies for their proactive role as scout. One CN metaphorically described this as “peeking” into the lives of residents; not intrusively, but with a sense of social awareness and concern to understand the lives of their community members:

For instance, a house that appears somewhat neglected. Who lives there now? And can we make a difference if you step inside? Literally peeking. Not peeking into their care but peeking into the overview [of their life]. Are there children? How large is the network? (Participant 47, RN, phase 1 – exploring).

Most CNs described the need to be proactive in individual and collective prevention. CNs stress the value of early identification of risk, underlining the benefits of a proactive rather than reactive approach.

After 1.5 years, we indeed have fewer crises to manage, because we have mapped out all older adults. We can identify risks earlier. (Participant 64, NP, phase 2 – deepening).

And identifying needs for prevention is not only for a client; it can also be given to a caregiver. One CN explained what she did as prevention for a caregiver: “*I have been working on this for years now, and as a CN, I see severely overburdened informal* caregivers. *I make them aware and try to protect them from the big pitfalls ahead [. . .]. So, I involve the carer in our collective project early* on.” (Participant 58, DN, phase 1 – exploring).

Finally, CNs emphasised that working with a holistic approach is important in collective prevention. Recognising the uniqueness of each context, they stressed the need to respond to people’s actual needs rather than rely on standardised solutions.

#### Motivator

CNs noted their responsibilities in individual prevention by stimulating individual behaviour change through home visits and lifestyle advice and in collective prevention by promoting participation in activities. In this context, the role of motivator goes beyond traditional motivational interviewing with a client. It involves a group-based approach by inspiring community members to participate in collective prevention initiatives and programmes. More specifically, NPs described that they transform initiatives to programmes. One NP shared an example.

You might say, ‘In this neighbourhood, we have noticed that alcohol problems play a significant role, along with loneliness. Shall we set something up to address that?’ We also organise a community lunch, where a large table is set up, and everyone is welcome to join. (Participant 67, NP, phase 2 – deepening).

CNs described their role as motivators, not only in relation to community members but also in reaching out to other key stakeholders in the neighbourhood. They often acted as professional coaches or, as one metaphor suggested, as a “spider in the web.” In this role, they link individuals to other professionals and community services.

**Figure 2.**
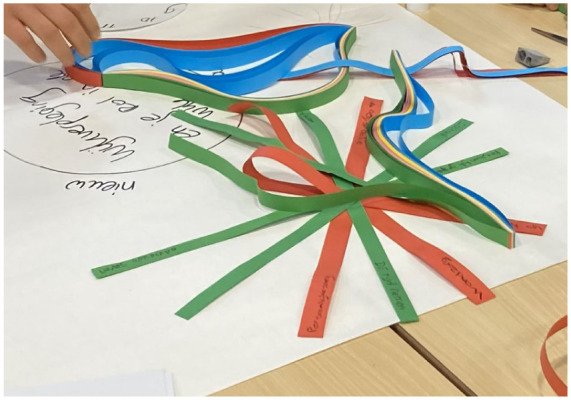
Phase 3 – deepening, “Spider in the web.”

As shown in Figure 2, they also integrate all types of tasks in their daily work. They give advice, provide wound care and personal care, use clinical nursing skills, and engage socially. They have personal conversations, manage administration, and give attention to others, all of which are captured in this “spider in the web” visualisation.

#### Facilitator

CNs often see themselves as connectors and bridge-builders, working across the boundaries of the medical, public health, and social care domains. As a facilitator, the CN’s expertise in both medical and social domains allows them to bridge the gap between medical nursing care and community development, translating complex health data into health and social interventions.

And at a certain point, we started making a lot of connections and thinking about how it could work. But you also notice that, through the contacts you build and by getting to know your neighbourhood well, you can sometimes also provide better care. This is because you have a clearer understanding of where someone can go with specific questions if you are aware of the available services. (Participant 44, DN, phase 1 – exploring).

CNs highlighted that they have dual expertise as nurses in a community, encompassing nursing, medical, and community care. This enables them to interpret public health data in ways that can be meaningful at the neighbourhood level, making practical interventions but also fostering collaboration between the various organisations active within the same community.

We also have knowledge of illness and health, which is more than what they [i.e., professionals from the social domain] have. We can, for example, see through research whether activities like exercise or sports can help prevent falls. They might know this as well, but we can complement that by sharing knowledge from the community with the social domain. So, I think you can complement each other, but I believe you do need each other. (Participant 68, NP, phase 2 – deepening).

Particularly NPs, among the CNs, emphasised their role as bridge-builders between the nursing and medical domains. In collective prevention they utilised this role in early detection but also in interprofessional collaboration with GPs and elderly care specialists.

#### Organiser

CNs described the need for collective prevention in the community and demonstrated many different competencies, such as taking initiative, creativity, and a keen eye for new opportunities. Through our observations, it became evident that many were well known locally, both by community members and by other (healthcare) professionals. They act autonomously and have an entrepreneurial spirit. The organiser role is shifting from organising nursing care to organising community initiatives.

Because that is sometimes the challenge in our conversations; getting people on our radar. So sometimes we simply take the initiative and walk in somewhere ourselves. [. . .] And that’s how it goes. (Participant 30, DN, phase 2 – deepening).

They also emphasised the importance of perseverance. Working in prevention takes commitment; setbacks are common, progress may be slow, and sustaining enthusiasm over time is essential.

We went to the municipality, and we are working on community living, but of course, that takes a long time. [. . .] And that, in turn, affects your own enthusiasm. And it is also about yourself. Of course, you know it’s a long-term effort. (Participant 51, DN, phase 2 – deepening).

CNs also reported that they often initiate and organise projects. This involves translating ideas into actionable steps, arranging funding, and bringing people together.

In the early stages of our project, people in a group said, ‘We’d like more connection in our communities. . . like creating a hangout spot for older people.’ [. . .] So, behind the scenes, we took it on ourselves. (Participant 6, DN, phase 2 – deepening).

NPs described a leadership role in initiating and sustaining preventive programmes. One NP shared an example of joining a fall-prevention initiative and transforming it from a general screening to a more comprehensive programme involving medical assessment, advice, and community care:

There was a fitness test day where seniors could assess their vitality, but no healthcare professionals were involved, so no one mentioned fall risk or high blood pressure. Last year, we added a preventive screening, and it reached a wide audience. It was really successful. That’s what we can do; bring in that broader picture and build that bridge between prevention and medical care. (Participant 66, NP, phase 2 – deepening).

Furthermore, they acted as coaches within community projects, encouraging local ownership and gradually stepping back when appropriate. The aim is not to control but to enable and facilitate.

Our senior fitness group is actually running really well. They are now doing everything themselves, and we do not really get involved anymore. . . (Participant 24, RN, phase 1 – exploration).

At the same time, the CNs underscored their responsibility to initiate meaningful conversations about prevention and explain the vision, even when met with hesitation or resistance from colleagues.

**Figure 3.**
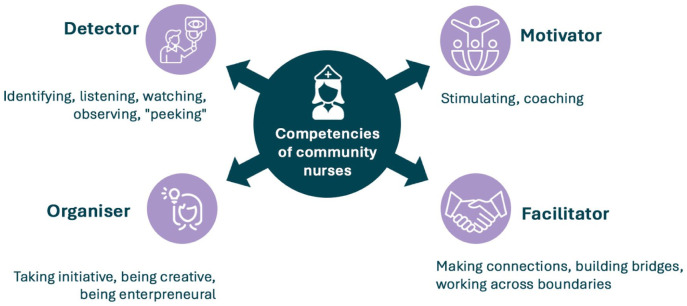
Summary of the roles and competencies of CNs in collective prevention.

A summary of the different roles and competencies is shown in Figure 3.

### Theme 3: Facilitating and Hindering Factors Influencing Collective Prevention Efforts

This theme identifies the key factors that either facilitate or hinder the implementation and sustainability of collective prevention efforts. While CNs gradually developed a deeper professional understanding of collective prevention over time, they encountered several systemic and team-related elements that influenced their ability to integrate these activities into their daily practice.

#### Facilitators

##### Driving Force

What consistently emerged from our conversations with the CNs and what we saw during the observations is that collective prevention does not simply unfold; it requires the spark and sustained effort of 1 or 2 driving forces within a team. These CNs often serve as frontrunners, laying the groundwork and guiding others in the team step-by-step into another area of their profession. Their efforts are enhanced when they proactively keep their team informed.

We have been guiding the team for two years now on what prevention is and what we exactly do. By now, everyone is convinced that it is incredibly important [. . .] we are involving the rest of the team along the way. (Participant 63, DN, phase 2- deepening).

The ability to engage and involve other CNs from the team in the process is essential. These initiators act as informal buddies to their colleagues who see limited need for collective prevention and for recently graduated colleagues and interns who have little to no experience in collective prevention. The CNs gradually expose their teams to the vision and ways of working of collective prevention. These driving forces often take along colleagues, starting by inviting them to participate in small engagements, such as offering coffee at events. These experiences then often lead to deeper involvement of their colleagues. As one participant said about her colleagues who lead a collective prevention project:

They took me along, and then they said: Oh, do you like it? Is this something for you? [. . .] At first, it was just coffee and tea, and then I started joining in. (Participant 42, (intern/RN, phase 2 – deepening).

##### Shared Vision on Collective Prevention

CNs emphasise the importance of a shared vision among all those working in the neighbourhood and interorganisational collaboration. A collective approach, underpinned by a coherent strategy across care providers, can reduce fragmentation and improve community impact. Because it minimises barriers between services, fosters direct communication among professionals, and ensures that clients receive more timely and coordinated support. Someone reflected on the profession of working in community care. In the past, there was a general practitioner (GP) and a CN in a neighbourhood. Then the work became more fragmented due to many specialisations in nursing. One participant suggested that we should return to that time.

If each of them [i.e. CNs] were responsible for a specific neighbourhood, we would be sorted. And if the GPs also joined in, we could create that essential combination of a GP and a CN in each area. (Participant 68, NP, phase 2 – deepening).

#### Barriers

##### Time and Workload

**Figure 4.**
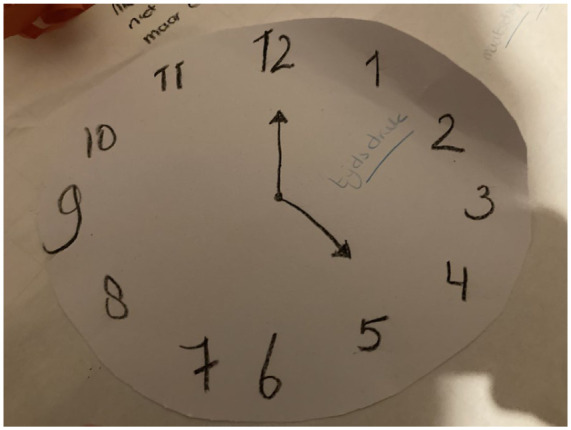
Phase 3 – validation, “time pressure.”

The urgent and complex needs of clients often take precedence, leaving little room for preventive initiatives. This challenge was even more present when team members were absent, forcing fewer CNs to manage the same workload. Under such types of conditions, the capacity to invest in collective prevention is, in fact, minimised: time. “*That plays a very big role. We even reached the point where we thought, can we even continue like this?*” (Participant 24, RN, phase 1 – exploring). Figure 4 illustrates how important CNs find time and the lack of time to work in collective prevention initiatives.

##### Balancing Between Direct Patient Care and Collective Prevention

For CNs, it is important to demonstrate for health insurers their productive hours in which CNs are active in direct client care such as wound care, personal care, and giving medication to clients.

No financial arrangements have yet been made with insurers to reimburse collective prevention activities; such activities are thus not yet funded. As a result, CNs are constantly navigating the tension between securing sufficient productivity by direct client care and engaging in other essential tasks, such as team meetings and collective prevention.

And we actually see this happening through the government as well. I mean, we are all assessed based on productivity, but we are not yet paid or recognised (also by the government) for engaging in collective prevention. (Participation 68, NP, phase 2 – deepening).

##### Resistance of Colleagues

Moreover, resistance of colleagues within teams can present a barrier. Key initiators often encounter scepticism from colleagues who view prevention as secondary to “real” nursing work, often referring to clinical nursing tasks as the “actual” work of nurses. Collective prevention is often not seen as the role of the CN. This results in the feeling among CNs that they had to defend their collective prevention activities. Some described how other team members seem to think that collective prevention is something that burdens the team, as it reduces the availability of the involved CNs for nursing care work.

And that is really part of our work that colleagues still have blinders on for. They come for the wound care, the showering, and the compression stockings. [. . .] Then they leave again. It is more about having that open mindset and the holistic approach, which should be much more [. . .]. That should be half of our work. (Participant 27, DN, phase 2 – deepening).

##### Fragmentation of Initiatives in Prevention

Another barrier is the fragmentation of initiatives in a community. The large number of initiatives, for example, the dancing group, the bingo night, billiards, and so on, often leaves community members unaware of available services, creating a situation where they cannot see the forest for the trees. As a result, the poor visibility of these initiatives does not help community members to take part in such initiatives. CNs who help initiate collective prevention need to involve the community members, other (health) professionals, and local initiatives to work together rather than doing something new again. Being in alignment with what is happening in a neighbourhood helps participation, referrals, and the sustainability of collective prevention.

There are often a lot of facilities and activities, but either it doesn’t fit, or people don’t know about it. (Participant 44, RN, phase 2 – deepening).

##### Sustainability in Initiatives

Sustainability is another recurring barrier for collective prevention efforts. It often hinges on the commitment of a few initiators. Without wider ownership among the team, these efforts may not survive when there are transitions in the team, such as the initiators leaving the team.

##### Nursing Education

Finally, CNs who participated in the study observed that newly qualified CNs are often unprepared for working in collective prevention; they had a hard time taking on the role of health promoters who can take initiative in collective prevention. Recently graduated CNs described their nursing education as clinically focussed, making them poorly prepared to navigate community preventive care. This resulted in other CNs needing to be available to help these new CNs integrate the vision and ways of working of collective prevention.

It also has to do with growing in your profession. When you graduate, you are so focused on the nursing situation and your goals and your interventions. That you are not yet dealing with the collective prevention at all and do not yet see it. [. . .] And you do not yet know your network very well. (Participant 66, NP, phase 2 – deepening).

## Discussion

This study examined CNs’ understanding of their role as health promoters while participating in a 3-year, collective prevention research project. CNs had the freedom to shape collective prevention in a way that suited the local context. They took the lead, deciding what was appropriate for their neighbourhood and community population.^28^

### The Concept of Prevention Described by CNs

The findings show a shift from individual prevention towards a broader collective approach. With CNs increasingly viewing themselves as proactive connectors who identify neighbourhood patterns, mobilise community members, and strengthen community health. They also face barriers and facilitators to fulfil this role. Lack of time, balance between patient care and doing collective prevention, and resistance from colleagues are seen as barriers. Facilitators share a vision for collective prevention and the fact that CNs are the driving force in group-based programmes.

In the study it was reflected that initially CNs focussed primarily on individual prevention. A reason for this is that, due to a history of product-thinking in which medicalised tasks were dominant, they also based their preventive efforts on that foundation. During the research, a shift toward a group-based approach occurred. CNs perceived this approach as more effective. The collective prevention initiatives are physically, socially, and mentally impactful in the lives of community members. Several other studies with group interventions have also identified social connectivity as a key mechanism to initiate and sustain physical intervention.^37^ In terms of collective prevention, CNs observed that they can engage community members more effectively at an earlier stage when collective prevention initiatives are being implemented. This enables a more proactive approach to delivering “the right care in the right place by the right professional.” This aligns with the WHO’s principles on community-based prevention and early health detection.^11,13^

### Roles and Competencies to Act as Health Promoters

The roles of CNs in collective prevention were examined in detail in this study. Although our sample size did not allow for deeper investigation of this, we did notice that the more experienced CNs seemed to emphasise the role of facilitator and bridge builder; this may be explained by their educational background, as collective prevention received more emphasis in earlier curricula than it does today. It is also observed internationally that there is not much focus on collective prevention in the nursing curricula. A call for a focus on health promotion and disease prevention in nursing curricula to get a community outcome is made in several other studies.^38,39^ It may also relate to their professional vision, as more experienced CNs tend to have a broader and deeper understanding of their role as health promoters. This is in line with Benner’s theory on the clinical competence of novice to expert. That has become foundational for many nursing curricula and development programmes.^40^ The theory describes how nurses develop skills and judgement through a combination of theoretical knowledge and practical experience over the years. This enables them to reason from impact rather than about the task.^40^

This study highlighted that several interventions are required to stimulate the implementation of collective prevention. Firstly, integrating education on the CN’s role into the basic curriculum.^41^ Secondly, providing workplace coaching on the competencies is advised.^42^ This could involve appointing “collective prevention ambassadors” within teams who coach colleagues on these roles. CNs reported that they found the focus groups, in which CNs exchanged ideas, were helpful, inspiring, and stimulating for implementing interventions. This effect could be facilitated by establishing a community of practice^43^ focussed on collective prevention.

### Facilitating and Hindering Factors in Collective Prevention

CNs face barriers and facilitators to fulfil this role. Facilitators include a shared vision and the CN being the driving force. However, many CNs experience a high workload and therefore do not have sufficient time for collective prevention interventions. Lack of time, balance between patient care and doing collective prevention, and resistance from colleagues are seen as primary barriers. The direct nursing care needs to be done first. The implementation of new working strategies takes time and effort in preventive healthcare because the results are not immediately seen.^44^ A recent systematic review showed that multicomponent preventive healthcare interventions may be the most effective strategy.^45^ For collective prevention, this implies simultaneously enhancing knowledge and motivation and removing physical and social barriers.

Another important barrier is that there is currently no funding for collective prevention in the Dutch healthcare system. CNs consistently report that participating in collective prevention activities influences their team productivity, as performance metrics are currently focussed on immediate client care rather than community-based initiatives. It requires that health and prevention be placed more prominently on the national policy agenda, ensuring that legislation and regulations support prevention rather than hinder it.^46,47^ At the moment, the costs of collective prevention are borne by the community care organisation, while the benefits are realised at a societal level. The structural underfunding of preventive care is not unique to the Dutch system; it is a global trend. Most OECD healthcare systems allocate less than 3% of their total health expenditure to prevention, prioritising curative services over population-based health interventions. Financing preventive healthcare measures needs a long-term and multi-domain policy.^47^ A clear political and policy vision is required to integrate collective prevention into the healthcare system.^48^

Thirdly, the focus on collective prevention is not just a novel way of working in the community care organisation Buurtzorg but is currently also being implemented by other organisations in the Netherlands.^49^

### Strengths and Limitations

Firstly, a strength of this study design is the longitudinal nature: the focus groups were held over a 3-year period, allowing insight into developments of the CNs’ understanding of prevention over time. Another strength is that we were able to focus on a single, nationwide community care organisation, allowing for a deep exploration of collective prevention. This singular focus provided an opportunity to understand how a community nursing care organisation facilitates or hinders collective preventive interventions.

Thirdly, this study included a broad range of CNs. They had various educational backgrounds and came from various regions across the Netherlands.

Finally, the first author had a dual position as both researcher and employee of the community care organisation. Her familiarity with the organisation and with community nursing can be considered a strength, for example, because she was able to connect effectively with the participants’ lived experiences.

### Limitations

While self-selection is inherent to most research, it is important to acknowledge that the participating teams were “frontrunners” who were already more engaged in or motivated to carry out collective prevention. These teams were voluntarily engaged in a collective prevention project. They were aware from the outset that they would participate in research and were willing to explore an innovation with uncertain outcomes, indicating an already entrepreneurial orientation.

Less attention could be given to differences in perspectives between CNs with various educational levels. The sample size did not allow for a comparative analysis based on the educational levels of the CNs. Although our sample size included diversity that allowed for studying diversity in perspectives, not all forms of diversity could be studied in detail due to the sample size of our study. Furthermore, this study focussed exclusively on CNs working in community nursing care.

While other healthcare and social care professionals play essential roles in collective prevention, our focus remained on the specific community nursing care perspective to gain interpretive depth. Future research should incorporate the perspectives of other (health) professionals and community members to further explore the impact of collective prevention interventions.

## Conclusion

In this study, conducted among community nurses, it became clear how CNs’ understanding of their role as health promoters evolved from individual prevention to collective prevention while participating in a long-term, collective prevention research project. CNs increasingly see themselves as health promoters with diverse roles, from detector and motivator to facilitator and organiser. Who not only provide care but also identify neighbourhood patterns, mobilise, and foster collaboration. Recognition of success by CNs is crucial for fostering programme ownership among community members. Proactivity, knowledge of local networks, and an entrepreneurial mindset are seen as essential, while time pressure, lack of funding, and insufficient preparation of newly qualified colleagues are major barriers. Nevertheless, the CNs emphasise that collective prevention is vital to the growing demand for care by strengthening health, social connection, and shared responsibility within communities. Strengthening collective prevention as a driver for sustainable and appropriate care requires its integration into national policy frameworks and education systems.

## Appendix 1

### Focus Group (Phase 1): District Nurses/Nurse Practitioners/Team Members Involved in the Collective Prevention Programmes

#### Welcome and Introduction

**Warm-Up Question**

What moment in the past year has stayed with you and made you happy regarding the prevention project?

**Prevention Level**

Could you describe how you think preventive care fits into the work of a district nurse? What do you think is needed to achieve this?

Why do you consider it important to focus on prevention in the community? What motivates you personally to engage in it?

**Participant Level**

What has been your experience so far with engaging with local residents? What challenges have you encountered? What causes these challenges? How could you solve them? What support do you need for this? What has gone well in establishing contact with residents? What lessons have you learned so far?

Which group of residents do you think you are currently reaching the most? [Healthy older adults, vulnerable older adults, volunteers, young people] Why do you think you are successful in reaching this group? Do you have insight into the motivation of these residents to participate in the project? What barriers do you experience in involving residents in the project?

In what ways are residents involved in the project’s activities? What different roles do residents play within the project? [participating, contributing ideas, organising, etc.] Could you explain how participants are encouraged to take on tasks in the organisation and development of the project? How is the community atmosphere now compared to a year ago? How has the interaction between community members changed since the start of the project? In what ways do you notice changes in the social life of community members?

How do you ensure that the project continues to align with the wishes and needs of the residents? How is the collaboration with residents progressing within this project? What role do practical matters such as location, timing, and summer breaks play?

Which target group are you currently not reaching but would like to reach? Could you explain why you think this group has not been reached yet? [Time investment, funding, knowledge].

**Project Level**

Together, we have defined several outcome measures and end goals, the most important being the improvement of well-being and the prevention of diseases. The 3 core outcomes measured in each project are perceived health, quality of life, and social participation. We would now like to discuss how you perceive these topics, your working methods, and whether they have changed since the project’s start.

To begin, what do you personally consider the most important outcomes/results of your project? When do you consider the project successful? [number of participants, increased participation, improved social connections, improved health/well-being, reduced loneliness] In what ways do you feel you are contributing to prevention through your project?

What do you consider important aspects for a project to run successfully? [Project elements, key figures, contextual factors like location/time, teamwork/collaboration].

What do you enjoy about working on your prevention project?

What do you find challenging or difficult about your work in the prevention project? Where or to whom can you turn for advice or support? How do you experience the role/support provided by Buurtzorg in this?

Where do you think improvements can still be made within your project? [outcome measures, involving residents, engaging colleagues, activities, content knowledge]

How do you envision the project’s future? What role do you see for yourself as a district nurse in this project? What role do you see for other (healthcare) professionals in this project? What opportunities do you see for further development of the project within the community? What opportunities do you see for expanding the project beyond the community?

**Programme Level**

Finally, we would like to discuss the Collective Prevention Programme and explore with you how you have experienced the guidance, information, and structure of the programme so far. Beforehand, we asked you to think about positive points and areas for improvement in the programme.

What do you appreciate about your participation in the Collective Prevention Programme? Knowledge, expertise, specific skills, funding, allocated hours? Expert guidance and support? Exchange with other projects: peer coaching and knowledge sharing?

What was different from what you expected in this learning/work programme?

Where do you see areas for improvement within the Collective Prevention Programme?

**Closing**

## Appendix 2

### Focus Group (Phase 2): District Nurses and Nurse Practitioners

**Theme:** Exploring what you perceive to be your roles, tasks, and responsibilities in community nursing regarding collective prevention. Additionally, identifying which knowledge and skills from your training have been helpful in this area.
1. **Opening Question**
a. What does collective prevention mean to you?
b. In what way are you involved in collective prevention? What is your role? What tasks do you perform? And how much time do you spend on it on average per week?
c. What would you say is the goal of collective prevention? What should it achieve?
d. What do you need to be able to carry out collective prevention in your community?
2. **Roles, Tasks, and Responsibilities in Community Nursing**
a. What do you believe is the role of community nursing when it comes to collective prevention?
b. Do you think collective prevention is part of your job in the community?
c. What tasks do you consider essential for implementing collective prevention in your community?
d. What qualities or skills do you think are necessary to engage in collective prevention?
e. What knowledge and skills do you need for collective prevention?
3. **Education and Training**
a. How are you equipped to engage in collective prevention in your community? Through your initial training? Through the organisation?
b. What resources are available to support collective prevention in your community?
4. **Conclusion**
a. Is there anything we missed or didn’t mention?
b. Summary of the discussion, highlighting key points.
c. Invitation for any final additions, comments, or questions.

## Appendix 3

### Focus Group (Phase 2) With Project Leaders and Team Members

**Theme:** exploring the role of the project leader in engaging the team members in a collective prevention program.
1. **Opening Question**
• How are you currently involved in the collective prevention project?
• Write this down on a post-it (preferably in 1 or 2 words); for example, enthusiastic, fragmented, unity, motivating, etc.
• Can you explain what you wrote? Why? What do you mean by it?
2. **Team Involvement**
• How do you feel about the team’s current involvement?
  ○ What’s going well?
  ○ What’s less effective?

- Perhaps you’ve also worked on enhancing team involvement. Could you share a bit about that?
  ○ How was it?
  ○ What went well?
  ○ What was challenging?

• Why do you think team involvement is important?
• How could involvement be improved?
• Question for community nurses not involved in the project: What’s your perspective on involvement in collective prevention?
3. **Raising Awareness Among Colleagues**
• What value does the team place on the collective prevention project?
• Could you explain this value? / Why do you place this value on it?
• What do you think; what value do individual team members place on the collective prevention project? / What have you heard from individual team members about their views on the project?
• What differences in perspective on collective prevention exist within the team, and what are they about?
• How have you tried to bring all team members on board with your vision for the collective prevention (project)?
  ○ What worked here?
  ○ What didn’t?

4. **What is Your Ideal Vision for Team Involvement in Collective Prevention?**
And specifically for this project, how do you see the future?
● What do you need to achieve this ideal vision?
● Who, what, where, and how?
5. **Closing**

## Appendix 4

### Themes For the World Café (Phase 3); Project Leaders

**Theme 1:** Community care and your role in the community; what is old en what is new?
**Theme 2:** How do you sustain a project?
**Theme 3:** What is community oriented prevention?

## References

[1] Ministerie van Volksgezondheid W en S. Passende zorg–Zorginstituut Nederland. 2023. Accessed May 27, 2025. https://www.zorginstituutnederland.nl/passende-zorg

[2] National Academies of Sciences, Engineering, and Medicine; National Academy of Medicine; Committee on the Future of Nursing 2020–2030; Flaubert JL Le Menestrel S Williams DR , et al. The nursing workforce. In: Flaubert JL , ed. The Future of Nursing 2020-2030: Charting a Path to Achieve Health Equity. National Academies Press; 2021: p. 21. Accessed May 29, 2025. https://www.ncbi.nlm.nih.gov/books/NBK573922/34524769

[3] Caron RM Noel K Reed RN Sibel J Smith HJ. Health promotion, health protection, and disease prevention: challenges and opportunities in a dynamic landscape. AJPM Focus. 2023;3(1):100167. doi:10.1016/j.focus.2023.100167 PMC10749873 38149078

[4] Lundin Gurné F Jakobsson S Lidén E Björkman I . District nurses’ perspectives on health-promotive and disease-preventive work at primary health care centres: a qualitative study. Scand J Caring Sci. 2023;37(1):153-162. doi:10.1111/scs.13100 35778918

[5] International Council of Nurses. Our nurses, our future. Featuring ICN’s new Charter for Change. 2023. Accessed January 2, 2025. https://www.icn.ch/sites/default/files/2023-07/ICN_IND_2023_Report_EN.pdf

[6] Sibbing H. De klepel en de klok. De Ottawa Charter. 2020; jaargang 20/nummer 4(Tijdschrift M&G):30,31.

[7] Casey D. Nurses’ perceptions, understanding and experiences of health promotion. J Clin Nurs. 2007;16(6):1039-1049. doi:10.1111/j.1365-2702.2007.01640.x 17518880

[8] Gomez Del Pulgar M Cuevas-Budhart MA Hernández-Iglesias S , et al. Best nursing intervention practices to prevent non-communicable disease: a systematic review. Public Health Rev. 2022;43:1604429. doi:10.3389/phrs.2022.1604429 PMC9516617 36189187

[9] Iriarte-Roteta A Lopez-Dicastillo O Mujika A , et al. Nurses’ role in health promotion and prevention: a critical interpretive synthesis. J Clin Nurs. 2020;29(21-22):3937-3949. doi:10.1111/jocn.15441 32757432

[10] Sassen B. Preventie en gezondheidsbevordering door verpleegkundigen. 2022. Accessed June 10, 2025. https://mijn.bsl.nl/preventie-en-gezondheidsbevordering-door-verpleegkundigen/20347410

[11] Nutbeam D Kickbusch I. Health promotion glossary. Health Promotion International. 1998;13(4):349-364.

[12] Wagemakers A Koelen MA Lezwijn J Vaandrager L. Coordinated action checklist: a tool for partnerships to facilitate and evaluate community health promotion. Glob Health Promot. 2010;17(3):17-28. Accessed June 12, 2025. https://journals.sagepub.com/doi/abs/10.1177/1757975910375166 10.1177/1757975910375166 21495437

[13] WHO. International Health Regulations – Third edition. 2005. Accessed June 12, 2025. https://www.who.int/publications/i/item/9789241580496

[14] DiClemente RJ Crosby R Kegler MC. Emerging Theories in Health Promotion Practice and Research: Strategies for Improving Public Health. John Wiley & Sons; 2002.

[15] Kennissynthese de wijkverpleegkundige van vandaag en morgen: rollen, samenwerking en deskundigheid van wijkverpleegkundigen. | Nivel. 2014. Accessed January 29, 2024. https://www.nivel.nl/nl/publicatie/kennissynthese-de-wijkverpleegkundige-van-vandaag-en-morgen-rollen-samenwerking-en

[16] van der Veen H de Leede-Brunsveld M . Collectieve preventie in de buurt. TVZ verpleegkd prakt wet. 2024;134(6):31-33. doi:10.1007/s41184-024-2389-0

[17] Veldhuizen J Schuurmans M Mikkers M Bleijenberg N. Advancing district nursing care through a learning healthcare system: a viewpoint on key requirements. Healthcare. 2024;12(24):24. doi:10.3390/healthcare12242576 PMC11727908 39766002

[18] Kuppen R de Leede M Lindenberg J van Bodegom D. Collective prevention of non-communicable diseases in an ageing population with community care. Int J Environ Res Public Health. 2023;20(4):3134. doi:10.3390/ijerph20043134 36833834 PMC9961588

[19] Jonas P Savigne EG Koster M Choonara I. Lessons from building a sustainable healthcare exchange between the Netherlands and Cuba. Int J Environ Res Public Health. 2022;19(18):11742. doi:10.3390/ijerph191811742 36142015 PMC9517359

[20] van Wijck F. Pleidooi voor wettelijk verankerde gezond-heids-doelen. DOQ. 2022. Accessed February 24, 2025. https://www.doq.nl/pleidooi-voor-wettelijk-verankerde-gezondheidsdoelen/

[21] Vlegel-Brouwer W Eelderink M Bussemaker J. Participatory action research as a driver for health promotion and prevention: a co-creation process between professionals and citizens in a deprived neighbourhood in the Hague. Int J Integra Care. 2023;23:13. Accessed February 24, 2025. https://ijic.org/articles/10.5334/ijic.7560 10.5334/ijic.7560 PMC10691282 38047119

[22] Bussemaker J Kramer A. Van weelde naar waarde. TSG Tijdschr Gezondheidswet. 2020;98(4):152-155. doi:10.1007/s12508-020-00283-7

[23] van de Vijver PL Wielens H Slaets JPJ van Bodegom D. Vitality club: a proof-of-principle of peer coaching for daily physical activity by older adults. Transl Behav Med. 2018;8(2):204-211. doi:10.1093/tbm/ibx035 29325113

[24] Bild E Pachana NA. Social prescribing: a narrative review of how community engagement can improve wellbeing in later life. J Community Appl Soc Psychol. 2022;32(6):1148-1215. doi:10.1002/casp.2631

[25] Marczak J Wistow G Fernandez JL. Evaluating social care prevention in England: challenges and opportunities. J Long-Term Care. 2019;2019:206-217. doi:10.31389/jltc.32

[26] Abma T Banks S Cook T , et al. Participatory Research for Health and Social Well-Being. Springer International Publishing; 2019. doi:10.1007/978-3-319-93191-3

[27] Polit DF Beck CT. Nursing Research: Generating and Assessing Evidence for Nursing Practice. 9th ed. Wolters Kluwer Health/Lippincott Williams & Wilkins; 2012.

[28] The Buurtzorg Model. Buurtzorg International. n.d. Accessed August 26, 2025. https://www.buurtzorg.com/about-us/buurtzorgmodel/

[29] Bernard HR. Research Methods in Anthropology: Qualitative and Quantitative Approaches. Sixth edition. Rowman & Littlefield; 2018.

[30] Brown J. A resource guide for hosting conversations that matter at The World Café | Better Evaluation. 2002. Accessed February 13, 2025. https://www.betterevaluation.org/tools-resources/resource-guide-for-hosting-conversations-matter-world-cafe

[31] Schiele H Krummaker S Hoffmann P Kowalski R. The “research world café” as method of scientific enquiry: combining rigor with relevance and speed. J Bus Res. 2022;140:280-296. doi:10.1016/j.jbusres.2021.10.075

[32] Clarke V Braun V. Thematic analysis. J Posit Psychol. 2017;12(3):297-298. doi:10.1080/17439760.2016.1262613

[33] Sim J Waterfield J. Focus group methodology: some ethical challenges. Qual Quant. 2019;53(6):3003-3022. doi:10.1007/s11135-019-00914-5

[34] Lincoln G. RWJF - Qualitative Research Guidelines Project | Lincoln & Guba | Lincoln and Guba’s Evaluative Criteria. 1985. Accessed February 11, 2025. http://www.qualres.org/HomeLinc-3684.html

[35] Donkoh S Mensah J. Application of triangulation in qualitative research. J Appl Biotechnol Bioeng. 2023;10(1):6-9. doi:10.15406/jabb.2023.10.00319

[36] Tong A Sainsbury P Craig J. Consolidated criteria for reporting qualitative research (COREQ): a 32-item checklist for interviews and focus groups. Int J Qual Health Care. 2007;19(6):349-357. doi:10.1093/intqhc/mzm042 17872937

[37] Burke S Carron A Eys M Ntoumanis N Estabrooks P. Group versus individual approach? A meta-analysis of the effectiveness of interventions to promote physical activity. J Sport Exerc Psychol. 2006;2:19-35.

[38] National Academies of Sciences, Engineering, and Medicine; National Academy of Medicine; Committee on the Future of Nursing 2020–2030 et al. Educating nurses for the future. In: Flaubert JL , ed. The Future of Nursing 2020-2030: Charting a Path to Achieve Health Equity. National Academies Press; 2021. p.141-142. Accessed December 18, 2025. https://www.ncbi.nlm.nih.gov/books/NBK573912/34524769

[39] Lasater K Atherton IM Kyle RG. Population health as a ‘platform’ for nurse education: a qualitative study of nursing leaders. Nurse Educ Today. 2020;86:104313. doi:10.1016/j.nedt.2019.104313 31923759

[40] Lamichhane G. Shaping professional nursing practice using Benner’s novice to expert theory. Br J Nurs. 2025;34(22): 1133-1137. doi:10.12968/bjon.2024.0387 41355560

[41] Bouwers A Broekman H Dobber J Eisenberg I den Hertog R Rutgers A. Opledingsprofiel bachelor nursing 2030. 2023. Accessed February 11, 2025. https://www.loov-hbov.nl/wp-content/uploads/2023/11/2023-10-30-BN2030.pdf

[42] Cannon-Bowers JA Bowers CA Carlson CE Doherty SL Evans J Hall J. Workplace coaching: a meta-analysis and recommendations for advancing the science of coaching. Front Psychol. 2023;14:1204166. doi:10.3389/fpsyg.2023.1204166 PMC10597717 37881215

[43] Albers M Gobbens RJJ Reitsma M Timmermans O a. a. MJ, Nies HLGR. Learning and innovation network in nursing: a concept analysis. Nurse Educ Today. 2021;104:104988. doi:10.1016/j.nedt.2021.104988 34246837

[44] Abdul Raheem Y . Unveiling the significance and challenges of integrating prevention levels in healthcare practice. J Prim Care Community Health. 2023;14:21501319231186500. doi:10.1177/21501319231186500 PMC10350749 37449436

[45] Heath L Stevens R Nicholson BD , et al. Strategies to improve the implementation of preventive care in primary care: a systematic review and meta-analysis. BMC Med. 2024;22(1):412. doi:10.1186/s12916-024-03588-5 39334345 PMC11437661

[46] Mierau JO van der Pol S Sandhu A Jansen DE. Performance assessment to improve public health systems. Bull World Health Organ. 2024;102(7):541-543. doi:10.2471/BLT.24.291543 38933475 PMC11197639

[47] Wouterse B Santos JV Hiligsmann M. Future directions for the economics of prevention. Expert Rev Pharmacoecon Outcomes Res. 2025;25(6):841-844. doi:10.1080/14737167.2025.2498665 40272784

[48] Masters R Anwar E Collins B Cookson R Capewell S. Return on investment of public health interventions: a systematic review. J Epidemiol Community Health. 2017;71(8):827-834. doi:10.1136/jech-2016-208141 28356325 PMC5537512

[49] De Buurt als Ecosysteem. Terug naar de toekomst van zorg–. n.d. Accessed November 17, 2025. https://debuurtalsecosysteem.nl/

